# Serotonin Mitigates ColdStress-Induced Damage in *Kandelia obovata* Through Modulating the Endogenous Melatonin- and Abscisic Acid Biosynthesis

**DOI:** 10.3390/ijms26041635

**Published:** 2025-02-14

**Authors:** Qiaobo Shan, Weicheng Liu, Xiaoxiao Ni, Min Li, Yifan Sun, Lixian Liao, Chunfang Zheng

**Affiliations:** 1 National and Local Joint Engineering Research Center of Ecological Treatment Technology for Urban Water Pollution, College of Life and Environmental Science, Wenzhou University, Wenzhou 325035, China; 17858366119@163.com (Q.S.); 22451335027@stu.wzu.edu.cn (X.N.); 23451039015@stu.wzu.edu.cn (M.L.); 23461042050@stu.wzu.edu.cn (Y.S.); 23451039019@stu.wzu.edu.cn (L.L.); 2 Zhejiang Key Laboratory of Coastal Biological Germplasm Resources Conservation and Utilization, Zhejiang Mariculture Research Institute, Wenzhou 325035, China; lwch80@126.com; 3 Wenzhou Key Laboratory of Marine Biological Genetics and Breeding, Zhejiang Mariculture Research Institute, Wenzhou 325000, China

**Keywords:** cold tolerance, serotonin, melatonin, abscisic acid, mangroves, RNA-seq

## Abstract

Endogenous melatonin (MEL) and abscisic acid (ABA) are involved in the adaptation of plants to environmental stresses. The application of exogenous serotonin (SER) to plants can enhance their tolerance to abiotic stress, such as cold. However, the mechanism associated with serotonin-mediated defense against cold-induced damage in mangroves is still poorly understood. In this study, we demonstrated that mangrove (*Kandelia obovata*) seedlings sprayed with 200 μmol·L^−1^ serotonin exhibited enhanced cold tolerance, as shown by reduced damage to leaves and loss of photosynthesis when exposed to low-temperature conditions. The mechanism associated with the cold adaptation of *K. obovata* seedlings upon treatment with serotonin was subsequently investigated by transcriptomic analysis. Serotonin treatment caused changes in differentially expressed genes (DEGs) involved in the regulation of melatonin (MEL) and ABA biosynthesis and defense responses against cold stress. Under low-temperature stress, serotonin-treated seedlings showed a significant increase in the endogenous levels of melatonin and ABA. By contrast, under normal growth conditions, *K. obovata* seedlings treated with serotonin displayed no substantial change in melatonin level, whereas ABA level significantly increased. These findings demonstrated that serotonin treatment might play an important role in the enhanced resistance to cold in *K. obovata* and that such an effect would depend on the activation of endogenous melatonin and ABA synthesis.

## 1. Introduction

Mangrove forest is considered the largest potential carbon stock because of its capacity to control greenhouse gas emissions and possess the capacity to reduce the rise in atmospheric CO_2_ levels [1,2]. Mangroves therefore play a significant role in regulating the climate [3]. According to Cohen et al. [4], global warming has led to the expansion of mangroves northward, especially in the northern hemisphere. With increasing global temperature, large increases in both the frequency and intensity of extremely cold events may become common in the regions where mangroves are distributed [5,6]. In these regions, frosts and freezes associated with severe cold weather events may reduce photosynthesis [7] and interrupt the process of resorption [8], leading to leaf scorch and bud withering [9], eventually killing the mangroves [10]. Therefore, understanding the biological mechanism underlying resistance to cold stress and improved cold tolerance can help mangroves to overwinter. Among the mangroves, *Kandelia obovata* is the most cold-tolerant species, and it is distributed mainly in the northernmost region of China [11].

Serotonin and melatonin are two major indoleamines derived from tryptophan, and both play important roles in plant growth and development [12]. In plants, serotonin and melatonin are produced from the precursor amino acid tryptophan via two primary pathways involving enzymes such as tryptophan decarboxylase (TDC), tryptophan hydroxylase (TPH), tryptamine 5-hydroxylase (T5H), and serotonin N-acetyltransferase (SNAT) [13]. Recent studies have demonstrated that serotonin can enhance the tolerance of plants to adverse conditions [14,15,16]. Notably, exogenous serotonin has been shown to improve cold tolerance by maintaining the osmotic potential balance of plant cells [17]. Our recent study has demonstrated that mangroves subjected to cold acclimation can enhance their resistance to cold via inducing the accumulation of endogenous serotonin, and the elevated level of serotonin can maintain a coordinated regulation of cold stress by reshaping the MEL/ROS/RNS redox network [18].

Cold resistance in plants has been shown to increase by treatment of the plants with exogenous abscisic acid [19,20,21]. Abscisic acid (ABA)-treated plants also show an increase in the level of endogenous ABA. ABA is an endogenous phytohormone that modulates plant response to cold stress [22]. In plants, the synthesis of ABA begins with β-carotene, which, through cyclization and hydroxylation, is converted to zeaxanthin [23]. Zeaxanthin is then converted to ABA through five sequential steps catalyzed by the enzymes zeaxanthin epoxidase (ZEP), neoxanthin synthase (NSY), 9-cis-epoxycarotenoid dioxygenase (NCED), alcohol dehydrogenase (ABA2), and abscisic aldehyde oxidase (AAO) [24,25,26]. In addition to ABA, another plant hormone that has also been shown to be associated with cold resistance is melatonin [27]. Melatonin and ABA can interact with each other, and such interaction is essential for the regulation of various physiological processes. For example, melatonin promotes ABA accumulation and limits non-stomatal water loss in watermelon under severe drought conditions, or induces *Arabidopsis* seed germination by promoting ABA catabolism [28,29,30]. Foliar application of melatonin and ABA can enhance cold tolerance in pumpkin, which is linked to the accumulation of both endogenous melatonin and ABA [25]. The increase in endogenous melatonin and ABA is a result of increased synthesis of these hormones rather than absorption of the exogenous source. The treated plants also display upregulated levels of *COMT1* and *NCED6*, key genes in the pathways of melatonin and ABA synthesis, respectively [25]. Since the application of ABA and melatonin can enhance cold resistance in mangroves, it is logical to expect a role for serotonin in ABA-mediated enhanced cold resistance in mangroves. In contrast to melatonin, the role of serotonin in plant stress is an evolving area of research that has for a long time been overshadowed by melatonin. In fact, many studies did not examine the role of serotonin and its connection to melatonin and ABA with respect to cold stress. To examine this hypothesis, we studied the effect of serotonin on cold tolerance in *K. obovata* by using a transcriptomic approach to identify serotonin-regulated gene sets. By analyzing massive transcriptome data, the physiological processes potentially related to cold tolerance were examined. The results should provide new insights into the mechanisms underlying serotonin-enhanced cold tolerance, providing valuable clues for enhancing the cold resistance of *K. obovata*.

## 2. Results

### 2.1. Serotonin Treatment Increases Cold Tolerance by Reducing Photosynthesis Loss

In order to confirm the ability of serotonin to induce tolerance to low-temperature stress, the impact of exogenous p-chlorophenylalanine (p-CPA), the most effective inhibitor of serotonin, on the growth and leaf photosynthesis of *K. obovata* seedlings under low-temperature conditions (6 °C/−3 °C) was determined. Seedlings that were sprayed with water exhibited abnormal growth characterized by wilting and desiccation, and in the case of p-CPA treatment, the leaves of the seedlings also turned dark brown, and the symptoms of frost damage in these seedlings were more obvious after the 3-day treatment (Figure S1). Seedlings treated with p-CPA exhibited decreased leaf *P*_n_, *G*_s_, *Φ*_PSII_, and *F*_V_/*F*_m_ by 66.19%, 56.35%, 25.81%, and 19.87%, respectively, when compared with those treated with just water (Figure S2). The result showed that p-CPA treatment could exacerbate damage caused by low-temperature stress. On the other hand, *K. obovata* seedlings treated with serotonin at different concentrations all displayed higher photosynthetic capacity compared to those treated with just water under low-temperature stress. The best effect was observed for seedlings that were treated with 200 μmol·L^−1^ of serotonin under low-temperature stress, and this was exemplified by their normal leaf morphology and increases in leaf *P*_n_, *G*_s_, *Φ*_PSII_, and *F*_V_/*F*_m_ by 168.42%, 74.37%, 78.30%, and 31.38%, respectively, compared with the water-treated seedlings (Figure S2).

### 2.2. Effect of Serotonin on Plant Growth and Photosynthesis of K. obovata

**Figure 1:**
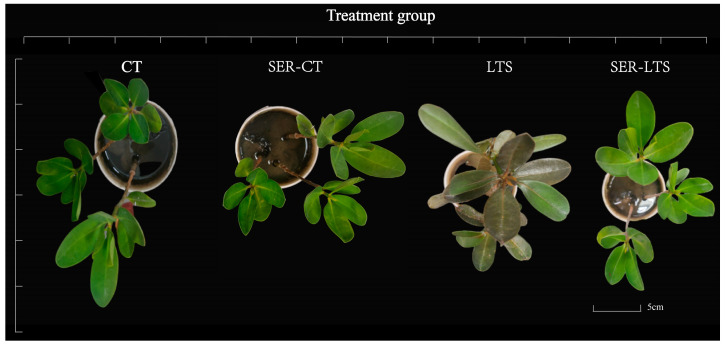
Serotonin pretreatment improves the cold resistance of *K. obovata* seedlings. Seedlings were grown under normal temperature conditions (25 °C/20 °C) without serotonin pretreatment (CT) or with serotonin pretreatment (SER-CT). Seedlings were grown under low-temperature conditions (6 °C/−3 °C) without serotonin pretreatment (LTS) or with serotonin pretreatment (SER-LTS).

In order to elucidate the role that serotonin might play in the regulation of cold tolerance in *K. obovata*, 200 μmmol·L^−1^ serotonin was selected for further experiments. Under normal temperature conditions (day/night temperature of 25 °C/20 °C), no obvious difference in morphology was observed between *K. obovata* treated with water (CT group) or serotonin (SER-CT group), as both exhibited similar growth and leaf texture. However, under low-temperature conditions (day/night temperature of 6 °C/−3 °C), the serotonin-treated seedlings (SER-LTS group) obviously retained normal leaf texture, while the seedlings treated with water (LTS group) displayed brownness in some of the leaves despite both groups of seedlings showing similar growth (Figure 1). From the morphological point of view, the result suggested that the benefit of serotonin pretreatment for *K. obovata* seedlings prior to cold stress could be the preservation of leaf texture.

**Figure 2:**
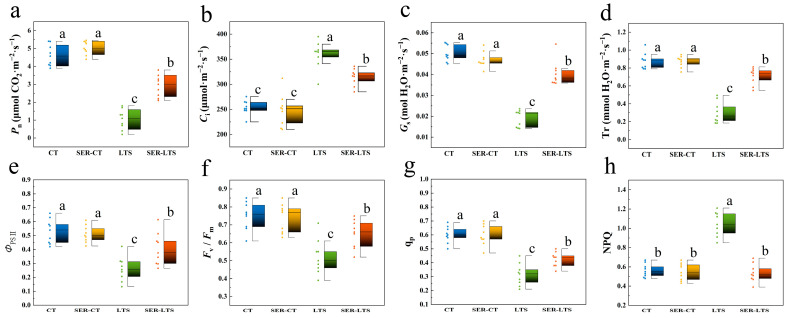
Effects of serotonin pretreatment on photosynthetic parameters of *K. obovata* seedlings grown under normal and low-temperature conditions. (**a**) *P*_n_: net photosynthetic rate; (**b**) *C*_i_: intercellular CO_2_ concentration; (**c**) *G*_s_: stomatal conductance; (**d**) *T*_r_: transpiration rate; (**e**) *Φ*_PSII_: actual efficiency of PSII; (**f**) *F*_v_/*F*_m_: maximum quantum efficiency of PSII; (**g**) q_p_: coefficient of photochemical fluorescence quenching; (**h**) NPQ: non-photochemical quenching. Error bars represent standard errors. Lowercase letters above the bars indicate differences among the groups that are of statistical significance (α = 0.05, LSD).

Low-temperature stress clearly disrupted the photosynthetic process of *K. obovata* seedlings as shown by the changes in texture and color of the leaves. The disruption of photosynthesis was subsequently evaluated by measuring various photosynthetic parameters of the leaves. For the seedlings from the LTS group, the leaf *P*_n_, *G*_s_, and *T*_r_ decreased by 66.19%, 56.35%, and 68.04%, respectively, while leaf *C*_i_ increased by 41.58% compared with the seedlings from the CT group (Figure 2). In contrast, seedlings from the SER-LTS group appeared to retain normal photosynthetic capacity, as shown by the significantly higher leaf *P*_n_, *G*_s_, *T*_r_, and *C*_i_ compared to those from the LTS group (Figure 2). The result of this experiment indicated that *K. obovata* seedlings sprayed with serotonin prior to exposure to low-temperature conditions could protect the seedlings against low-temperature stress by preventing the disruption of photosynthesis.

Chlorophyll fluorescence analysis techniques have been used to shed further light on the protective mechanism of enhancing tolerance to cold damage inflicted on mangroves [31]. *Kandelia obovate* from the LTS group exhibited decreased leaf *Φ*_PSII_, *F*_v_/*F*_m_, and q_p_, but increased leaf NPQ compared with those from the CT group (Figure 2). However, for the SER-LTS group of seedlings, leaf *Φ*_PSII_, *F*_v_/F_m_, and q_p_ increased by 78.30%, 31.38%, and 29.61%, respectively, while leaf NPQ was reduced by 48.56%, compared with the seedlings from the LTS group, and these differences were statistically significant (Figure 2). Again, no statistical difference in leaf *Φ*_PSII_, *F*_v_/*F*_m_, q_p_, and NPQ between the CT group and SER-CT group. The results provided further support that the application of serotonin to *K. obovata* seedlings prior to cold stress acted as protection against cold-induced damage, allowing the seedlings to grow and function normally.

### 2.3. Differentially Expressed Genes (DEGs) Associated with Serotonin Pretreatment

To identify genes that displayed differential expression upon pretreatment with serotonin and cultivation under normal and cold temperature conditions, leaves collected from the four different treatment groups of *K. obovata* seedlings were subjected to transcriptomic analysis. A total of 12 cDNA libraries were constructed and sequenced, yielding a total of 576,914,828 raw reads. High-quality clean reads accounted for 98.13% of the total reads, and the average Q30 (0.1% error rate) was 95.26%, suggesting that the clean reads sequenced via RNA-seq were high-quality with a low mismatch rate. By filtering out low-quality reads and adapters, high-quality and sufficient clean reads were obtained (Table S1). The results of Pearson’s correlation coefficient analysis reflected the reproducibility among the biological samples (Figure S3a). The principal component analysis (PCA) of the transcriptome data showed a distinct separation between the LTS and SER-LTS groups. The first principal component (PC1) and the second principal component (PC2) explained 76.2% of the total variance (Figure S3b), suggesting that serotonin and low-temperature stress had a major impact on the gene expression profiles. As shown in Figure S4, 4131 differentially expressed genes (DEGs) were identified from the SER-CT versus CT groups, and 2745 of these were up-regulated and 1386 were down-regulated, while 5312 DEGs were identified from the SER-LTS versus LTS groups, 2152 of which were up-regulated and 3160 of which were down-regulated (Figure S4).

#### 2.3.1. Functional KEGG Annotation

**Figure 3:**
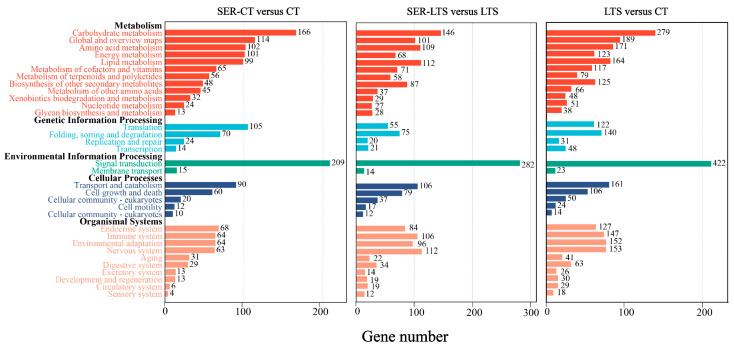
KEGG functional classification of DEGs identified from the comparison of different treatment groups of *K. obovata* seedlings.

To study the molecular mechanisms underlying the cold tolerance in *K. obovata* seedlings, a total of 14,938 DEGs from three comparison groups (SER-CT versus CT, SER-LTS versus LTS, LTS versus CT) were assigned to five major categories: metabolism, genetic information processing, environmental information processing, cellular processes, and organismal systems using the Kyoto Encyclopedia of Genes and Genomes (KEGG) pathway database. The largest number of DEGs were assigned to the “signal transduction” subgroup within the environmental information processing category, followed by “carbohydrate metabolism” and “transport and catabolism,” which are within the metabolism category and cellular processes, respectively (Figure 3). The results suggested that these related pathways might be important pathways involved in the serotonin-induced response of *K. obovata* seedlings to environmental changes.

#### 2.3.2. KEGG Enrichment Analysis for DEGs

To further explore the potential pathways involved in serotonin-induced cold tolerance, DEGs from SER-CT versus CT groups, SER-LTS versus LTS groups, and LTS versus CT groups across relevant biological pathways were analyzed using the KEGG database, and the results indicated significant enrichment in 9, 10, and 12 pathways (*p* < 0.05), respectively. The DEGs were significantly enriched in photosynthesis, carbohydrates, amino acids, and nucleic acid metabolism. Changes in the expression levels of genes related to these processes were verified by RT-qPCR. These selected genes encode rubisco accumulation factor (Raf), Mg-protoporphyrin IX methyltransferase (CHLM), geranylgeranyl diphosphate reductase (GGDR), phosphoribulokinase (PRK), transketolase (TRK), mitochondrial carrier family (MCF), peroxisomal membrane protein (PMP), and long chain acyl-CoA synthetase (LCACs). The relative expression levels of these genes, as indicated by their transcript levels, corresponded to the FPKM values, which increased in seedlings following serotonin pretreatment and were either kept under normal or low-temperature conditions, confirming the reliability of the transcriptome data (Figure S5).

**Figure 4:**
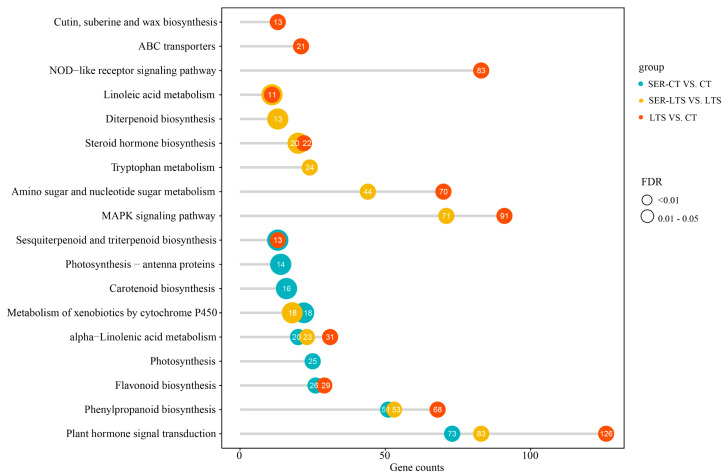
KEGG enrichment analysis of DEGs derived from the comparison of *K. obovata* seedlings from treatment groups. Different colors represent different group comparisons, and the size of the circle represents different false discovery rate (FDR) intervals.

In the SER-CT versus CT and SER-LTS versus LTS comparisons, four pathways were found to be the same based on the types of DEGs enriched, and these pathways included phenylpropanoid biosynthesis, alpha-linolenic acid metabolism, plant hormone signal transduction, and metabolism of xenobiotics by cytochrome P450 (Figure 4). In the SER-CT versus CT group comparison, carotenoid biosynthesis (ko00906) was significantly enriched (*p* < 0.05, Figure 4), while in the SER-LTS versus LTS group comparison, tryptophan metabolism (ko00380) was significantly enriched (*p* < 0.05) (Figure 4). However, in the LTS versus CT group comparison, both tryptophan metabolism (ko00380) and carotenoid biosynthesis (ko00906) were not significantly enriched. Taken together, the result suggested that tryptophan and carotenoid metabolism were highly correlated with serotonin pretreatment.

#### 2.3.3. Dynamic Transcriptome Analysis

**Figure 5:**
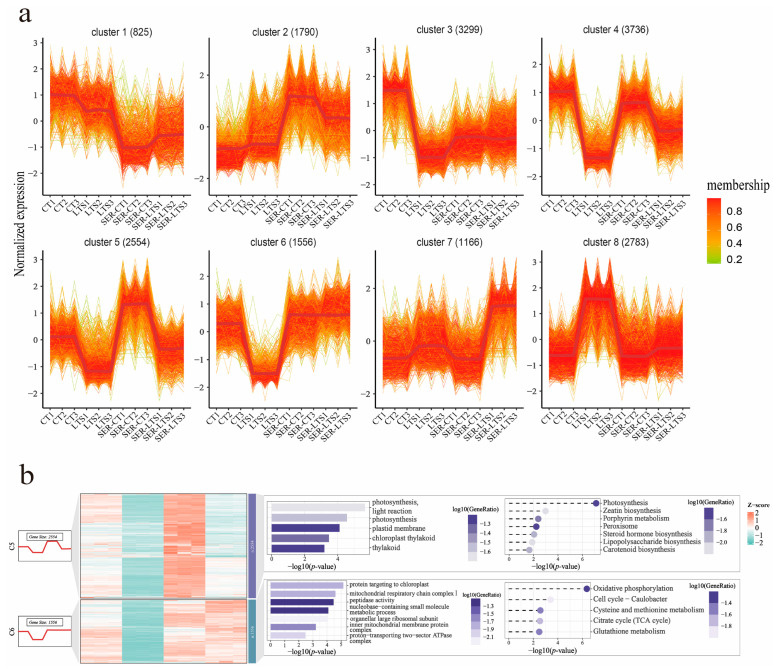
Clustering for gene expression based on the k-means method. (**a**) Calculated unique expression patterns of genes based on the expression levels of all genes. The number above each plot indicates the number of genes per cluster. (**b**) KEGG and GO enrichment results for the genes of Cluster 5 and Cluster 6.

From the transcriptome analysis, we calculated the expression of each gene in the different treatment groups and clustered the genes with similar expression patterns in the four treatment groups using the k-means clustering method [32,33]. Eight gene clusters with similar expression trends were obtained. Among them, the genes in Cluster 5 showed an up-regulated trend in the case of serotonin treatment followed by exposure to normal and low-temperature conditions, and the genes in Cluster 6 remained highly expressed after serotonin treatment (Figure 5a). The genes in both clusters showed a down-regulated trend under low-temperature stress (Figure 5a). KEGG and gene ontology (GO) enrichment analysis were then performed for the genes of these two clusters (clusters 5 and 6). The genes in Cluster 5 were also significantly enriched for carotenoid biosynthesis (Figure 5b), consistent with the KEGG analysis results (Figure 4), indicating that genes related to carotenoid metabolism tended to be upregulated by serotonin pretreatment.

#### 2.3.4. DEGs Enriched in Melatonin Biosynthesis Pathways

**Figure 6:**
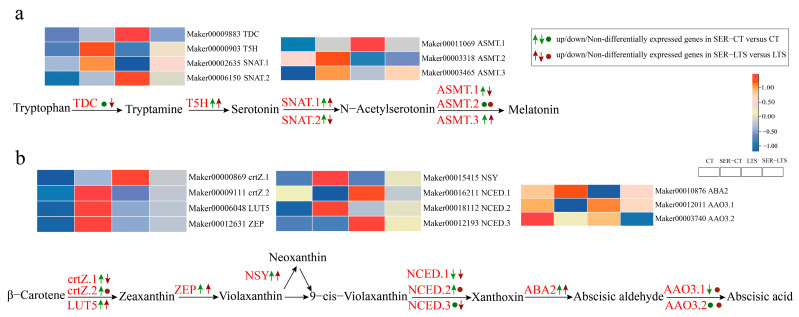
Heatmap of DEGs involved in melatonin and ABA biosynthesis in cold-stressed *K. obovata* leaves. (**a**) Melatonin biosynthesis. (**b**) ABA biosynthesis. Genes with a foldchange greater than 1 were marked as upregulated, genes with foldchange less than −1 were marked as downregulated, and others were non-differentiated.

The KEGG pathway classification and functional enrichment results indicated an effect of serotonin on the genes involved in the biosynthesis of melatonin (Figure 4). Genes such as cytochrome P450, TDC, T5H, and SNAT have been identified as being involved in melatonin biosynthesis [34,35]. In the melatonin biosynthesis pathway, the gene (*Maker00009883*) encoding L-tryptophan decarboxylase (TDC) [EC:4.1.1.105] was downregulated, as revealed by the SER-LTS versus LTS group comparison. The expression of tryptamine 5-hydroxylase (T5H) represented by *Maker00000903* [EC:1.14.-.-] was the highest in the SER-CT group and lowest in the LTS group (Figure 6a). Two genes (*Maker00002635* and *Maker00006150)* encoded aralkylamine N-acetyltransferase (SNAT) [EC:2.3.1.87], and three genes (*Maker00011069*, *Maker00003318*, and *Maker00003465*) encoded acetylserotonin O-methyltransferase (ASMT) [EC:2.1.1.4]. An upregulated expression was detected for the *Maker00002635* (SNAT.1), *Maker00006150* (SNAT.2), *Maker00011069* (ASMT.1), and *Maker0003465* (ASMT.3) genes in the SER-CT versus CT group comparison, whereas in the SER-LTS versus LTS group comparison, only *Maker00002635* (SNAT.1) and *Maker0003465* (ASMT.3) displayed upregulated expression, while *Maker00006150* (SNAT.2) and *Maker00011069* (ASMT.1) both exhibited downregulated expression (Figure 6a). These results indicated that serotonin pre-treatment could positively affect the genes of melatonin biosynthesis.

#### 2.3.5. Gene Expression of ABA Biosynthesis

The biosynthesis of abscisic acid (ABA) can be traced to carotenoid, and the key enzymes involved in ABA biosynthesis are beta-carotene 3-hydroxylase (crtZ), beta-ring hydroxylase (LUT5), zeaxanthin epoxidase (ZEP), and neoxanthin synthase (NSY) [22]. According to KEGG enrichment analysis, the genes coding for these enzymes all exhibited changes in expression in the comparison of the SER-CT versus CT and SER-LTS versus LTS groups (Figure 5b). In the ABA biosynthesis pathway, the SER-CT versus CT group comparison identified upregulated expression for the genes encoding beta-carotene 3-hydroxylase (crtZ) [EC:1.14.15.24] (*Maker00000869* and *Maker00009111*), whereas both the SER-CT versus CT and SER-LTS versus LTS group comparisons identified upregulated expression for the genes encoding beta-ring hydroxylase (LUT5) [EC:1.14.-.-] (*Maker00006048*), zeaxanthin epoxidase (ZEP) [EC:1.14.15.21] (*Maker00012631*), and neoxanthin synthase (NSY) [EC:5.3.99.9] (*Maker00015415*) (Figure 6b), with the expression of ZEP being the highest among the genes in the SER-CT group. Three DEGs involved in ABA biosynthesis were found, and these were *Maker00016211*, *Maker00018112*, and *Maker00012193*. The DNA sequences of these three markers were found to contain the coding sequence for a putative 9-cis-epoxycarotenoid dioxygenase (NCED) [EC:1.13.11.51]. *Maker00016211* was downregulated according to the SER-CT versus CT and SER-LTS versus LTS group comparisons, whereas *Maker00018112* was upregulated as shown by the SER-CT with CT group comparison. *Maker00010876*, a gene encoding a xanthoxin dehydrogenase (ABA2) [EC:1.1.1.288], was upregulated in both SER-LTS versus LTS and SER-LTS versus LTS group comparisons. Although there are two genes coding for abscisic-aldehyde oxidase (AAO3), *Maker00012011* and *Maker00003740*, only *Maker0001211* was identified as being downregulated in the SER-CT versus CT group comparison, with *Maker00003740* showing no change in expression (Figure 6b). These findings suggested that ABA could be an essential positive regulator of serotonin-mediated cold tolerance in *K. obovata* seedlings.

### 2.4. Effect of Serotonin on Biosynthesis of Melatonin and ABA in K. obovata Seedlings

#### 2.4.1. Melatonin Biosynthesis

**Figure 7:**
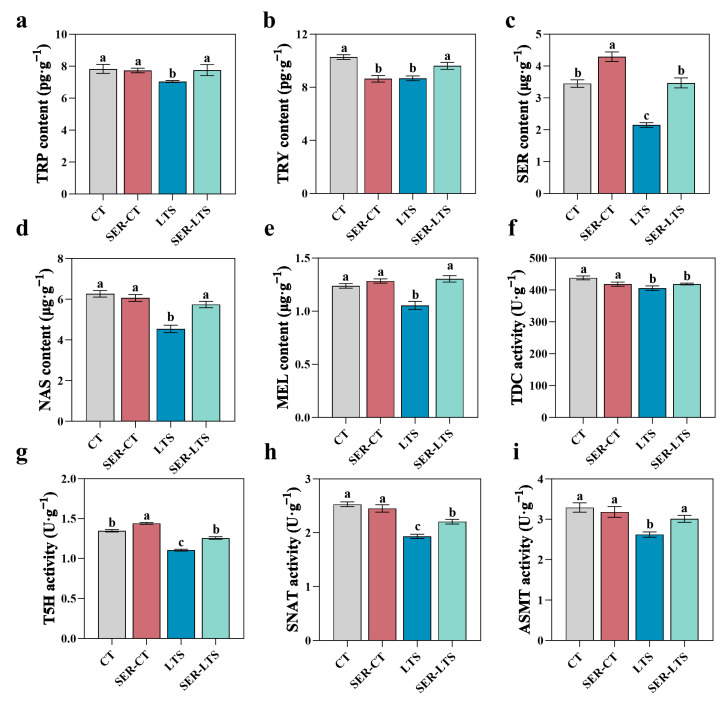
Changes in the levels of precursors and intermediates and the effect of serotonin pretreatment on the activity levels of four key enzymes involved in the synthesis of serotonin in *K. obovata* seedlings. (**a**) Content of tryptophan (TRP). (**b**) Content of tryptamine (TRY). (**c**) Content of serotonin (SER). (**d**) Content of N-acetyl-serotonin (NAS). (**e**) Content of melatonin (MEL). (**f**) Activity of decarboxylase (TDC). (**g**) Activity of tryptamine-5-hydroxylase (T5H). (**h**) Activity of serotonin-N-acetyltransferase (SNAT). (**i**) Activity of N-acetyl-serotonin-methyltransferase (ASMT). Error bars represent standard errors, and the lowercase letters above the bar indicate differences of statistical significance (α = 0.05, LSD) among the groups.

In order to further investigate the role of serotonin in melatonin-induced tolerance to cold stress, the classic pathway of melatonin biosynthesis (tryptophan/tryptamine/serotonin/N-acetylserotonin/melatonin) in plants was measured. Seedlings from the LTS group exhibited reduced biosynthesis of melatonin, as demonstrated by the reduced leaf TRP, TRY, SER, NAS, and MEL contents of 10.11%, 15.56%, 49.90%, 29.03%, and 14.82%, respectively, compared to seedlings from the CT group (Figure 7a–e). However, for the seedlings from the SER-LTS group, the impact of low temperatures on melatonin biosynthesis was mitigated since the leaf contents of TRP, TRY, SER, NAS, and MEL in these seedlings increased by 10.19%, 16.00%, 61.34%, 26.46%, and 23.63%, respectively, compared to those from the LTS group. Under normal temperature conditions, seedlings receiving serotonin pre-treatment (SER-CT group) exhibited increased serotonin content in their leaves by 24.41%, while their TRY content was decreased by 15.88% compared to seedlings without serotonin pretreatment (CT group). No statistical differences in the contents of TRP, NAS, and MEL were observed between the absence and presence of serotonin pretreatment followed by exposure to normal temperature conditions (Figure 7a,d,e).

The activities of leaf TDC, T5H, SANT, and ASMT decreased by 7.39%, 15.76%, 23.62%, and 20.39%, respectively, for seedlings from the LTS group compared with seedlings from the CT group (Figure 7f–i). However, such losses of activities were mitigated upon pretreatment with serotonin prior to low-temperature exposures (SER-LTS group), resulting in the enhancement of the corresponding enzyme activities: 3.23%, 13.80%, 14.13%, and 14.94% for TDC, T5H, SANT, and ASMT, respectively, in comparison with control seedlings kept under low temperatures (LTS group) (Figure 7f–i). No significant difference was observed for leaf TDC, T5H, SANT, and ASMT activities between the CT and SER-CT groups of seedlings, suggesting that under normal temperature conditions, serotonin pretreatment offered no real advantages to *K. obovata* seedlings.

#### 2.4.2. ABA Biosynthesis

**Figure 8:**
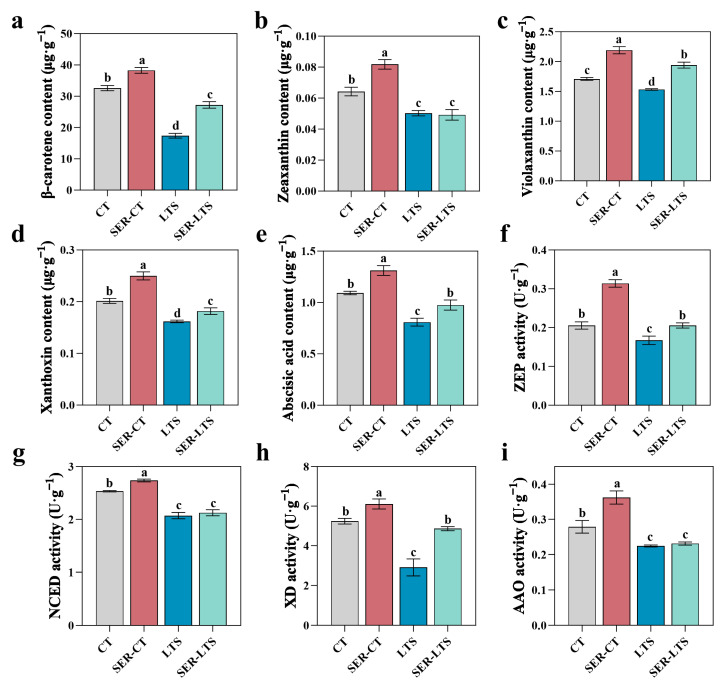
Effect of serotonin pretreatment on the content of intermediates and the activities of enzymes involved in the conversion of β-carotenoids to ABA. (**a**) Content of β-carotenoids. (**b**) Content of zeaxanthin. (**c**) Content of violaxanthin. (**d**) Content of xanthixin. (**e**) Content of abscisic acid. (**f**) Activity of zeaxanthin epoxidase (ZEP). (**g**) Activity of 9-cis-epoxycarotenoid dioxygenase (NCED). (**h**) Activity of xanthoxin dehydrogenase (XD). (**i**) Activity of aldehyde oxidase (AAO). Error bars represent standard errors. and the lowercase letters above the bars indicate differences of statistical significance (α = 0.05, LSD) among the groups.

ABA biosynthesis represents a minor branch of the carotenoid pathway [36,37]. To investigate whether serotonin directly regulates the ABA biosynthetic pathway, the impact of serotonin pretreatment on the conversion of carotenoids into ABA in *K. obovata* seedlings was investigated. Compared to seedlings from the CT group, seedlings from the LTS group displayed decreases in the contents of β-carotene, zeaxanthin, violaxanthin, xanthoxin, and ABA of 46.66%, 25.17%, 10.25%, 19.66%, and 25.97%, respectively (Figure 8a–e). Seedlings from the SER-LTS group showed reduced loss in these hormones except for zeaxanthin, with both groups having a similar zeaxanthin content. The contents of β-carotene, violaxanthin, xanthoxin, and ABA were 56.66%, 26.57%, 12.29%, and 20.55%, respectively—higher in the SER-LTS group compared with the LTS group—although these contents were still significantly lower compared to the SER-CT group (Figure 8a–e). Significant differences in the contents of β-carotene, zeaxanthin, violaxanthin, xanthoxin, and ABA were detected between the CT and SER-CT groups, confirming that serotonin pretreatment promoted the synthesis of these hormones.

Seedlings from the LTS group showed a significant decrease in ZEP and XD activity levels, with as much as 18.26% (ZEP) and 44.52% (XD) reduction compared to seedlings from the CT group. In contrast, seedlings from the SER-LTS group had 22.79% more ZEP activity and 67.21% more XD activity than the seedlings from the LTS group, while no statistical difference was detected in the NCED and AAO activity levels between the two groups (Figure 8f–i). Significant differences in the activity levels of ZEP, NCED, XD, and AAO were also detected between the CT group and the SER-CT group. Consistent with the previous results, these data suggested that serotonin may contribute to increased carotenoid metabolism and ABA biosynthesis.

### 2.5. Correlation Analysis of Photosynthetic Characteristics, ABA Biosynthesis and Melatonin Biosynthesis

**Figure 9:**
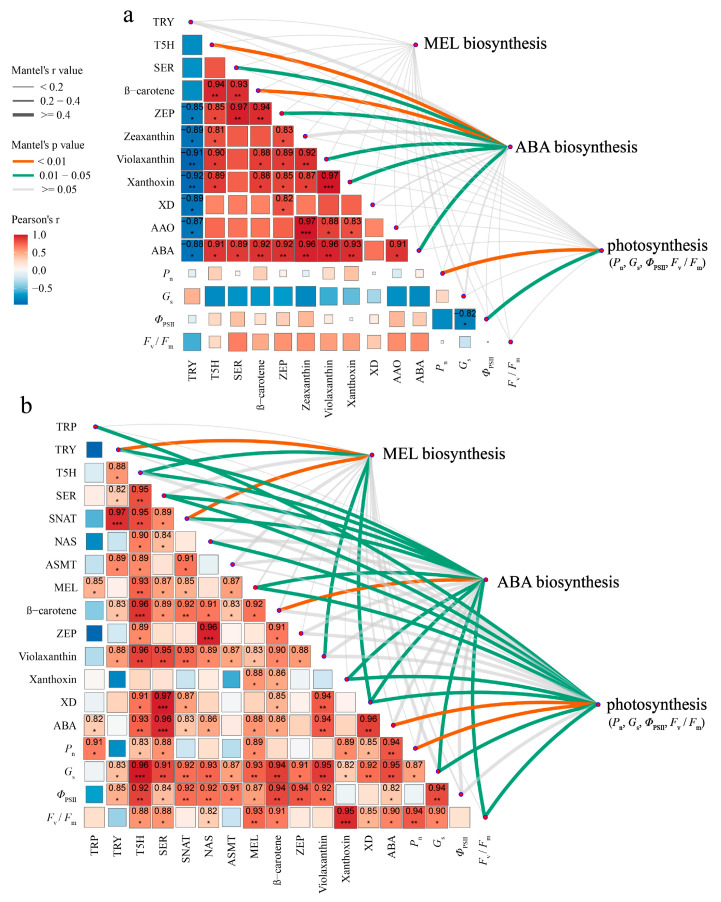
Ggcor correlation combination diagram and regulatory network of key indicators that displayed significant changes under the effects of serotonin pretreatment and temperatures. (**a**) Correlation among melatonin biosynthesis, abscisic acid biosynthesis, and key physiological indexes under normal conditions with serotonin pretreatment. (**b**) Correlations between melatonin biosynthesis, abscisic acid biosynthesis, and photosynthesis parameters under low temperatures with serotonin pretreatment. Rows and columns correspond to the physiological indexes, and each cell contains the corresponding correlation and *p*-value (* indicates *p*-value < 0.05, ** indicates *p*-value < 0.01, *** indicates *p*-value < 0.001). Pearson’s R-values are color-coded according to the color legend. The curve width corresponds to the Mantel’s r statistic for the correlations between the different biosynthesis, photosynthesis, and key physiological indexes. The curve color corresponds to the Mantel’s *P* statistic for the correlations between biosynthesis, photosynthesis, and key physiological indexes. The greater the correlation coefficient of Mantel’s r value, the smaller the Mantel’s *p* value, indicating that the greater the impact of physiological indexes on different biosynthesis and photosynthesis.

To investigate the potential regulatory mechanism of serotonin associated with cold tolerance, the Mantel test was performed to reveal this interaction between photosynthetic characteristics (*P*_n_, *G*_s_, *Φ*_PSII_, and *F*_v_/*F*_m_), factors in ABA biosynthesis (β-carotene, zeaxanthin, ZEP, violaxanthin, NCED, xanthoxin, XD, AAO, and ABA), and factors in melatonin biosynthesis (TRP, TDC, TRY, T5H, SER, SNAT, NAS, ASMT, and MEL). A significant response in leaf ABA biosynthesis manifested by the changes in T5H activity and b-carotene content (Mantel’s *p* < 0.01) was detected in the seedlings from the CT and SER-CT groups. Moreover, correlation analysis of ABA biosynthesis found a significant, positive correlation between the xanthoxin and violaxanthin contents (*p* < 0.01) and between the AAO activity and zeaxanthin content (*p* < 0.001). There was a significant positive relationship (*p* < 0.01) between ABA content and β-carotene, zeaxanthin, violaxanthin, xanthoxin contents, and ZEP activity. Meanwhile, ABA biosynthesis was found to be significantly (*p* < 0.05) correlated with each of the following: ABA content, T5H activity, AAO activity, and serotonin content (Figure 9). For the seedlings from the SER-LTS group, the biosynthesis of leaf melatonin was significantly related to the TRY content and SNAT activity (Mantel’s *p* < 0.01) and to the T5H and XD activities and violaxanthin content (Mantel’s *p* < 0.05). ABA biosynthesis was significantly related to β-carotene content (Mantel’s *p* < 0.01); TRY, SER, NAS, MEL, and xanthoxin contents; T5H, SNAT, and XD activities; and *G*_s_ and *F*_v_/*F*_m_ (Mantel’s *p* < 0.05). Additionally, leaf photosynthesis was significantly related to ABA content and *P*_n_ activity (Mantel’s *p* < 0.01); TRP, SER, NAS, and MEL contents; XD activity; and *P*_n_, *G*_s_, and *F*_v_/*F*_m_ (Mantel’s *p* < 0.05) (Figure 9). According to the Pearson’s correlation test, the melatonin content was significantly positively correlated with T5H activity, *F*_v_/*F*_m_, and *G*_s_ (*p* < 0.01) and was significantly correlated with ABA biosynthesis and photosynthesis under low-temperature stress. These results suggested that enhanced cold tolerance provided by serotonin pretreatment could be attributed mainly to the regulation of melatonin and ABA syntheses and to the photosynthesis capacity of *K. obovata* seedlings.

## 3. Discussion

### 3.1. Serotonin Conferred Improved Cold Tolerance in K. obovata Seedlings

Serotonin plays an important role in defense-related responses [38]. The application of serotonin to a plant can improve its tolerance to abiotic stress by improving its photosynthetic parameters [14,15,39]. Low-temperature-stress is known to induce a loss of photosynthetic activity in plants by destroying PSI and PSII [40]. Previous studies have revealed that low temperatures can decrease the photosynthetic capacity of *K. obovata* seedlings as a result of reduced leaf *P*_n_, *Φ*_PSII_, and *F*_v_/*F*_m_ [41,42]. We have previously reported that endogenous serotonin can induce cold tolerance in *K. obovata* by upregulating photosynthetic carbon assimilation capacity [43]. In the present study, we found that low-temperature stress could prevent the PSII photochemistry of the seedlings from reaching the maximal quantum yield, leading to reduced photosynthetic rate, stomatal conductance, and transpiration rate, consistent with what our previous study has shown [44]. The protective role against cold stress provided by the application of serotonin could be seen via the mitigation of severe leaf wilting. Furthermore, when the activity of serotonin was blocked by the inhibitor p-CPA, the protective role of serotonin was compromised, providing direct evidence for the role of serotonin in the protection of *K. abovate* against cold stress. There seemed to be an optimum concentration of serotonin associated with the protection of seedlings against cold stress when applied externally. Our data suggested that 200 μmol·L^−1^ could be the optimal concentration, as demonstrated by the maximum enhancement in photosynthetic activity and modulation of other gas exchange parameters (*G*_s_, *C*_i_, and *T*_r_) (Figure 2). At the molecular level, the expression levels of a series of genes coding for proteins/enzymes related to photosynthesis were found to be markedly induced by serotonin treatment, including genes that code for Raf, CHLM, GGDR, PRK, TRK, MCF, PMP, and LCACs (Figure S5).

### 3.2. Serotonin Enhances Cold Tolerance via Altering Melatonin Biosynthesis of K. obovata Seedlings

Recent studies have found that externally applied melatonin might play a role in the enhancement of cold tolerance in plants by altering endogenous melatonin biosynthesis [45,46]. Melatonin is a downstream product of serotonin that can improve photosynthesis through various means, including increasing the photosynthetic pigment content, regulating the stomatal aperture, and downregulating the chlorophyll degradation genes [47,48,49]. Likewise, in *K. obovata* seedlings, endogenous melatonin biosynthesis was also enhanced by treatment with serotonin, which at the same time also helped to maintain an optimal level of photosynthesis capacity to protect the functions of PSI and PSII. This might suggest that enhanced melatonin levels within the seedlings might be the key factor in the maintenance of photosynthetic capacity, and this could be the basis of enhanced cold resistance observed for the *K. obovata* seedlings under the influence of serotonin pretreatment. Repeated exposure of *K. obovata* seedlings to a gradual decrease in temperature followed by a short period of recovery can lead to a gradual increase in the endogenous serotonin level within the seedlings [18]. Under such a treatment scheme, the level of cytochrome P450 monooxygenase was found to increase, and since this enzyme catalyzes the hydroxylation of tryptamine to serotonin during the synthesis of serotonin [34,50], the gradual increase in endogenous serotonin is linked to enhanced biosynthesis of serotonin. In our study, the transcription levels of the cytochrome P450 monooxygenase gene and the key gene T5H for serotonin biosynthesis were strongly upregulated after pre-treatment with serotonin under low and normal temperature conditions (Figure 4 and Figure 6), consistent with the observed changes in endogenous serotonin content (Figure 7c). Regarding melatonin biosynthesis, serotonin treatment significantly increased the endogenous serotonin content but did not induce the accumulation of melatonin under normal temperature conditions. However, at low temperatures, pretreatment with serotonin significantly improved the accumulation of both serotonin and melatonin within the seedlings (Figure 7e), suggesting that low temperatures somehow induced the endogenous serotonin to stimulate the biosynthesis of melatonin in *K. obovata* through pretreatment with serotonin. Furthermore, in the pathway of melatonin biosynthesis, 24 DEGs were identified, including *Maker00002635* (SNAT.1), *Maker00006150* (SNAT.2), *Maker00011069* (ASMT.1), *Maker00003318* (ASMT.2), and *Maker0003465* (ASMT.3). The expressions of *Maker00002635* (SNAT.1) and *Maker0003465* (ASMT.3) tended to increase and was significantly induced by serotonin pretreatment prior to cold temperature stress, which also led to increased SNAT and ASMT activities (Figure 6a and Figure 7h,i). The results indicated that both *Maker00002635* (SNAT.1) and *Maker0003465* (ASMT.3) in *K. obovata* might play a key role in the endogenous synthesis of melatonin.

Under low temperatures, the expression of T5H was upregulated only in *K. obovata* seedlings that were given serotonin pretreatment (Figure 7g). Thus, we could speculate that the accumulation of endogenous serotonin was partly a result of its own synthesis. In plants, TDC and T5H are two key enzymes that catalyze the synthesis of serotonin from tryptophan. However, no significant change in either the expression or activity of TDC was detected in seedlings following serotonin pretreatment (Figure 6a and Figure 7f). This might explain the sudden decrease in tryptamine content in *K. obovata* seedlings under normal temperature conditions following serotonin pretreatment (Figure 7b). The physiological impact of serotonin pretreatment was investigated in terms of the changes in activity levels of T5H, SNAT, and ASMT as well as the contents of TRP, TRY, SER, NAS, and MEL, all of which exhibited a significant increase when serotonin treatment was given under low-temperature conditions (Figure 9b). This suggested that serotonin pre-treatment conducted under low-temperature stress was the instigating factor for such a physiological change, and the increased activity levels of T5H, SNAT, and ASMT effectively increased the flux of tryptophan toward the formation of melatonin.

### 3.3. Serotonin Improves Cold Tolerance by Positively Regulating ABA Biosynthesis Pathway of K. obovata

Serotonin has been demonstrated to play a mediating role in ABA-mediated regulation of salt stress tolerance [51]. Extensive studies have supported the conclusion that endogenous ABA can significantly enhance plant defense against cold-induced damage [22,52,53,54]. However, the molecular mechanisms mediated by ABA under low-temperature stress remain unclear. In our study, transcriptome analysis showed that low-temperature exposure led to a significant reduction in *Maker00010876* (ABA2) and *Maker00003740* (AAO) expression in *K. obovata* seedlings (Figure 6b) and decreased activity levels for the ABA synthetic enzymes ZEP, XD, and AAO (Figure 6b and Figure 8f,h,i), ultimately resulting in reduced synthesis of ABA (Figure 8e), and this was strongly associated with a reduction of photosynthesis in the cold-stressed *K. obovata* (Figure 9). Consistent with these changes, transcriptome analysis showed that the expression of genes involved in the ABA biosynthesis pathway was significantly activated by serotonin pre-treatment under normal and low-temperature conditions (Figure 5b). Of these, the expression of *Maker00006048* (LUT5), *Maker00012631* (ZEP), *Maker00015415* (NSY), and *Maker00010876* (ABA2) in both SER-CT and SER-LTS plants was significantly upregulated (Figure 6b), indicating that serotonin pre-treatment induced the conversion of β-carotene to ABA. Meanwhile, activity assays revealed a significant enhancement in the activities of ZEP and XD (both involved in ABA biosynthesis) in serotonin-pretreated *K. obovata* seedlings, whether under normal or low-temperature conditions (Figure 8f,h), consistent with increased ABA levels in these seedlings (Figure 8e). The results showed that serotonin pre-treatment modulated the ABA biosynthesis pathway under normal and low temperatures, with ZEP and XD emerging as critical enzymes within this pathway. Furthermore, the level of ABA in the serotonin-pretreated seedlings was 34.0% higher under normal-temperature conditions compared with low-temperature conditions (Figure 8e). Thus, another possible reason for enhancing cold tolerance in *K. obovata* seedlings may be the effective enhancement of endogenous ABA synthesis under normal temperatures mediated by serotonin applied as a pretreatment, which allowed the accumulated ABA to exert its impact during the transition to cold temperature conditions.

## 4. Materials and Methods

### 4.1. Plant Materials and Growth Conditions

One-year-old *K. obovata* seedlings were collected from Quanzhou Bay Mangrove Nature Reserve (24° 47′ N, 118° 37′ E), Fujian, China. These seedlings were planted in plastic pots (20 cm diameter and 30 cm height), each filled with 3 kg of intertidal soil, and with three seedlings planted per pot. The pots were kept in a glass greenhouse with a daytime temperature of 24 ± 5 °C and a nighttime temperature of 18 ± 5 °C. All pots containing the seedlings were watered every 2 days to keep the soil saturated with water.

### 4.2. Pretreatment of Seedlings and Exposure to Cold Stress

The pots of seedlings were transferred to a plant growth chamber and kept there for seven days. All seedlings were subjected to a 12 h/12 h light–dark period with a temperature of 25 °C and 20 °C for the light and dark periods, respectively. During the light period, the seedlings were illuminated with a photosynthetically effective radiation of 400 μmol·m^−2^·s^−1^. The relative humidity of the chamber was 60 ± 5%. The seedlings were divided into three groups: the control group, the serotonin group, and the serotonin inhibitor group. The control group was sprayed with water only (50 mL per seedling), while the serotonin group was sprayed with different concentrations (100, 200, 300, and 500 μmol·L^−1^) of serotonin (50 mL per seedling), while the serotonin inhibitor group was sprayed with 500 μmol·L^−1^ DL-4-chlorophenylalanine (p-CPA, the most effective inhibitor of serotonin), also 50 mL per seedling. The spraying was performed twice a day, once at 9:00 a.m. and again at 9:00 p.m., over a three-day period. After the three-day treatment, these seedlings were transferred to a cold chamber (LGZD-250Y, Hangzhou Lvbo Instrument Co., Ltd., Hangzhou, China) for another three days with a temperature of 6 °C during the 12 h light period and −3 °C during the 12 h dark period. After the three-day period, the third fully expanded leaf from the seedling was used to measure the net photosynthetic rate (*P*_n_) and actual efficiency of PSII (*Φ*_PSII_).

The serotonin concentration that gave the best growth based on leaf morphology, net photosynthetic rate (*P*_n_), stomatal conductance (*G*_s_), actual efficiency of PSII (*Φ*_PSII_), and maximum quantum efficiency of PSII (*F*_v_/*F*_m_), was selected for subsequent experiments. A similar setup of seedlings was prepared, involving a total of 24 seedlings divided into four groups, with 6 seedlings per group. After the initial seven days of growth in the glass greenhouse, the pots of seedlings were transferred to the same growth chamber with the temperatures of light and dark periods set at 25 °C and 20 °C, respectively. Two groups were kept as controls by spraying with just water, whereas the other two groups were sprayed with serotonin using the concentration that gave the best photosynthetic capacity. After spraying, all seedlings were kept at the same temperatures (25 °C/20 °C) for three days. After that, one control (CT) group and one serotonin-treated (SER-CT) group were then kept at the same temperatures, whereas the remaining control (LTS) group and serotonin-treated (SER-LTS) group were transferred to a cold chamber with temperatures set at 6 °C and −3 °C for the light and dark periods, respectively. After three days, the third fully expanded leaf of the seedling was immediately used to determine physiological parameters. The experiments were performed in a randomized complete block design, with three replicates for each group.

### 4.3. Gas Exchange Parameters and Chlorophyll Fluorescence Parameters Assay

The gas exchange parameters of the third fully expanded leaves were measured using a portable photosynthesis system (LI-6400, LI-COR Inc., Lincoln, NE, USA) at 9:30–11:30 a.m. The leaf chamber was equipped with a red and blue LED light source and a photosynthetically active radiation (PAR) of 400 μmol·m^−2^·s^−1^. The concentration of CO_2_ and air velocity were maintained at 390 ± 10 μmol·mol^−1^ and 500 μmol·s^−1^, respectively.

Chlorophyll fluorescence parameters of the third fully expanded leaves, which included the actual efficiency of PSII (*Φ*_PSII_), the maximum quantum efficiency of PSII (*F*_v_/*F*_m_), the photochemical fluorescence quenching coefficient (q_p_), and the non-photochemical quenching (NPQ), were measured with a pulse modulated fluorometer (Hansatech Instruments, UK) as described in Liu et al. [31] and calculated according to Yuan et al. [55].

### 4.4. Enzyme Activity Assays

About 0.1 g of the third fully expanded leaves was cut into small pieces and placed in a grinding tube containing 0.9 mL of phosphate buffer solution (pH 7.4) while kept in an ice bath and then ground into a homogenate using a SCIENTZ-48 high-throughput tissue grinder. The homogenate was then centrifuged at 1500× *g*/4 °C for 15 min, and the supernatant was subjected to enzyme activity and metabolite assays. Enzyme-linked immunosorbent assay (ELISA) kits (Shanghai Youxuan Biotech, Shanghai, China) were used to determine the activities of zeaxanthin epoxidase (ZEP), 9-cis-epoxycarotenoid dioxygenase (NCED), xanthoxin dehydrogenase (XD), aldehyde oxidase (AAO), decarboxylase (TDC), tryptamine-5-hydroxylase (T5H), serotonin-N-acetyltransferase (SNAT), N-acetyl-serotonin-methyltransferase (ASMT), and the content of tryptophan (TRP), tryptamine (TRY), serotonin (SER), N-acetyl-serotonin (NAS), melatonin (MEL), β-carotenoids, zeaxanthin, violaxanthin, xanthixin, and abscisic acid (ABA). All assays were performed according to the manufacturer’s instructions, and the Epoch Microplate Spectrophotometer (BioTek, Winooski, VT, USA) was used for spectrophotometric measurement where necessary. The catalog number and specific name of the kits are shown in Table S3.

### 4.5. RNA-Seq Analysis

A total of 12 independent RNA-Seq libraries were prepared from the third fully expanded leaves taken from the four groups (CT, LTS, SER-CT, SER-LTS) of *K. obovata* seedlings, with three biological replicates for each group. First, total RNA was extracted from the leaves using the TRIzol reagent (Invitrogen, Carlsbad, CA, USA) according to the manufacturer’s protocol. RNA purity and concentration were evaluated using the NanoDrop 2000 spectrophotometer (Thermo Scientific, Waltham, MA, USA). RNA integrity was assessed using the Agilent 2100 Bioanalyzer (Agilent Technologies, Santa Clara, CA, USA). Next, RNA-Seq libraries were constructed from the extracted RNA using a VAHTS Universal V6 RNA-Seq Library Prep Kit according to the manufacturer’s instructions. After that, the libraries were sequenced on an Illumina Novaseq 6000 platform. The genome sequence and annotation data of *K. obovata* were obtained from the Genome Sequence Archive database (https://ngdc.cncb.ac.cn/gsa/browse/CRA002395, accessed on 11 October 2023) [56]. Paired-end reads were aligned to the *K. obovata* genome using HISAT2 (V. 2.1.1) [57]. The reads were quantified using featureCounts 2.16.0 [58], and the DESeq2 1.46.0 software was used to calculate differentially expressed genes based on the number of original reads in the sample [59]. Gene expression levels were normalized using the transcripts per million (TPM) method and fragments per kilobase of exon model per million mapped fragments (FPKM) method. The protein sequences were annotated using EGGNOG (http://eggnog-mapper.embl.de/, accessed on 10 November 2023) and GO (http://geneontology.org/, accessed on 10 November 2023). Enrichment analysis was conducted using the R package clusterProfiler 4.2.3. The KEGG classification histogram was plotted using the program from https://www.bioinformatics.com.cn (last accessed on 16 August 2024) [60]. Transcriptome expression trend analysis was performed using the R package ClusterGVis (junjunlab/ClusterGVis: One-step to Cluster and Visualize Gene Expression Matrix (github.com), https://github.com/junjunlab/ClusterGVis, accessed on 15 September 2024).

### 4.6. Quantitative Real-Time PCR (RT-qPCR)

Total RNA was extracted from frozen leaves using TRIzol reagent (Invitrogen, Carlsbad, CA, USA) following the manufacturer’s instructions. The extracted RNA was used to synthesize the cDNA using the PrimeScript II First Strand cDNA Synthesis Kit (TaKaRa, Shiga, Japan). RT-qPCR was performed on a MA-6000 real-time PCR system using PerfectStart Green qPCR SuperMix (Transgen, Beijing, China). The sequences of the primers used in RT-qPCR are listed in Table S2. The relative mRNA level of each gene was calculated using the 2^−∆∆CT^ method and normalized to 18S rRNA [44,61]. The melt curve plot of all genes as shown in Figure S6.

### 4.7. Statistical Analysis

Data were calculated using Origin 2022 (OriginLab, Northampton, MA, USA) and presented as means ± standard deviations (SDs) from three replicates. Data were analyzed using one-way ANOVA (Duncan’s test). Statistical significance was considered at the *p* < 0.05 level. Correlation analysis and the mantel test for photosynthesis and the biosynthesis of melatonin and ABA were performed using R 4.3.1.

## 5. Conclusions

Cold stress can have a detrimental impact on the growth and proper functioning of plants by causing a loss of photosynthetic capacity, which could translate to physical damage to leaf tissues. We have demonstrated that external application of serotonin protected mangrove seedlings from cold stress-induced damage to leaf tissue by preventing the loss of photosynthetic capacity. The underlying factors that contributed to such mitigation appeared to be a concurrent increase in both melatonin and ABA levels in the leaves, which probably occurred as a result of increased expression and activities of key enzymes involved in the biosynthetic pathways of both hormones. However, the precise trigger that led to such a phenomenon has yet to be identified, and further study is needed to clarify this issue. Nevertheless, the findings of this study may suggest that manipulating the endogenous levels of melatonin and ABA may serve as potential strategies of serotonin to enhance the cold tolerance of plants in cold regions.

## Data Availability

The data presented in this study are available on request from the corresponding author.

## Supplementary Materials

The following supporting information can be downloaded at: https://www.mdpi.com/article/10.3390/ijms26041635/s1.

## References

[1] Choudhary B., Dhar V., Pawase A.S. (2024). Blue Carbon and the Role of Mangroves in Carbon Sequestration: Its Mechanisms, Estimation, Human Impacts and Conservation Strategies for Economic Incentives. J. Sea Res..

[2] Sahari M.S.I., Mohd Razali N.A., Redzuan N.S., Shah A.M., Awang N.A., Lee L.H., Juahir H., Muhammad Nor S.M. (2024). Carbon Stock Variability of Setiu Lagoon Mangroves and Its Relation to the Environmental Parameters. Glob. Ecol. Conserv..

[3] Fu C., Li Y., Zeng L., Zhang H., Tu C., Zhou Q., Xiong K., Wu J., Duarte C.M., Christie P. (2021). Stocks and Losses of Soil Organic Carbon from Chinese Vegetated Coastal Habitats. Glob. Change Biol..

[4] Cohen M.C.L. (2021). Effects of the 2017-2018 Winter Freeze on the Northern Limit of the American Mangroves, Mississippi River Delta Plain. Geomorphology.

[5] Coldren G.A., Langley J.A., Feller I.C., Chapman S.K. (2019). Warming Accelerates Mangrove Expansion and Surface Elevation Gain in a Subtropical Wetland. J. Ecol..

[6] Osland M.J., Day R.H., Michot T.C. (2020). Frequency of Extreme Freeze Events Controls the Distribution and Structure of Black Mangroves (*Avicennia germinans*) near Their Northern Range Limit in Coastal Louisiana. Divers. Distrib..

[7] Zheng C., Liu W., Qiu J., Huang L., Huang X., Chen S. (2013). Comparison of Physiological Characteristics of *Kandelia Obovata* at Different Ages in Winter in the Northernmost Mangrove Transplanted Area of China. Acta Ecol. Sin..

[8] Ellis W.L., Bowles J.W., Erickson A.A., Stafford N., Bell S.S., Thomas M. (2006). Alteration of the Chemical Composition of Mangrove (*Laguncularia racemosa*) Leaf Litter Fall by Freeze Damage. Estuar. Coast. Shelf Sci..

[9] Wang W., You S., Wang Y., Huang L., Wang M. (2011). Influence of Frost on Nutrient Resorption during Leaf Senescence in a Mangrove at Its Latitudinal Limit of Distribution. Plant Soil.

[10] Zheng C., Ye Y., Liu W., Tang J., Zhang C., Qiu J., Chen J. (2016). Recovery of Photosynthesis, Sucrose Metabolism, and Proteolytic Enzymes in *Kandelia obovata* from Rare Cold Events in the Northernmost Mangrove, China. Ecol Process.

[11] Xu X., Hou C., Shen Y. (2023). The Complete Mitochondrial Genome of the *Kandelia Obovata* Sheue, H.Y.Liu & J.W.H.Yong (Rhizophoraceae). Mitochondrial DNA Part B.

[12] Erland L.A.E., Murch S.J., Reiter R.J., Saxena P.K. (2015). A New Balancing Act: The Many Roles of Melatonin and Serotonin in Plant Growth and Development. Plant Signal. Behav..

[13] Gao Y., Chen H., Chen D., Hao G. (2023). Genetic and Evolutionary Dissection of Melatonin Response Signaling Facilitates the Regulation of Plant Growth and Stress Responses. J. Pineal Res..

[14] Akcay U.C., Okudan N. (2023). Exogenous Serotonin Improves Drought and Salt Tolerance in Tomato Seedlings. Plant Growth Regul..

[15] Pontes C.V.S., Dos Santos A.H.A., Lopes L.K.C., Barbosa M.A.M., Bajguz A., Da Silva Lobato A.K. (2024). Exogenous Serotonin and 24-Epibrassinolide Boost Root Protection and Suppress Oxidative Damages Occasioned by Severe Water Deficit in Soybean Seedlings. J. Plant Growth Regul..

[16] Zahra N., Kausar A., Abdelghani H.T.M., Singh S., Vashishth D.S., Bachheti A., Bachheti R.K., Husen A. (2024). Serotonin Improves Plant Growth, Foliar Functions and Antioxidant Defence System in Ethiopian Mustard (*Brassica carinata* A. Br.). S. Afr. J. Bot..

[17] He H. (2021). Study on the Mechanism of Exogenous Serotonin Improving Cold Tolerance of Rapeseed (*Brassica napus* L.) Seedlings. Plant Growth Regul..

[18] Li J., Zhang H., Yue D., Chen S., Yin Y., Zheng C., Chen Y. (2024). Endogenous Serotonin Induced by Cold Acclimation Increases Cold Tolerance by Reshaping the MEL/ROS/RNS Redox Network in *Kandelia obovata*. J. For. Res..

[19] Chen Q., Liu P., Li Z., Zheng Q., Zhou H., Liu J., Cao P., Fang M. (2024). Accumulated Endogenous Abscisic Acid Contributes to the Cold Tolerance of Pre-Planted Cultivated Tobacco. Plant Mol. Biol. Rep..

[20] Cuevas J.C., López-Cobollo R., Alcázar R., Zarza X., Koncz C., Altabella T., Salinas J., Tiburcio A.F., Ferrando A. (2008). Putrescine Is Involved in *Arabidopsis* Freezing Tolerance and Cold Acclimation by Regulating Abscisic Acid Levels in Response to Low Temperature. Plant Physiol..

[21] Wang Y., Ding S., Chen Z., Wang X., Jiang Q., Zhao J., Duan B., Xi Z. (2023). Transcriptomic Analysis Provides Insights into the Abscisic Acid Mediates Brassinosteroid-Induced Cold Resistance of Grapevine (*Vitis vinifera* L.). Plant Growth Regul..

[22] Singh A., Roychoudhury A. (2023). Abscisic Acid in Plants under Abiotic Stress: Crosstalk with Major Phytohormones. Plant Cell Rep..

[23] An J.-P., Xu R.-R., Liu X., Su L., Yang K., Wang X.-F., Wang G.-L., You C.-X. (2022). Abscisic Acid Insensitive 4 Interacts with ICE1 and JAZ Proteins to Regulate ABA Signaling-Mediated Cold Tolerance in Apple. J. Exp. Bot..

[24] González-Guzmán M., Abia D., Salinas J., Serrano R., Rodríguez P.L. (2004). Two New Alleles of the *Abscisic Aldehyde Oxidase 3* Gene Reveal Its Role in Abscisic Acid Biosynthesis in Seeds. Plant Physiol..

[25] Guo Y., Yan J., Su Z., Chang J., Yang J., Wei C., Zhang Y., Ma J., Zhang X., Li H. (2021). Abscisic Acid Mediates Grafting-Induced Cold Tolerance of Watermelon via Interaction with Melatonin and Methyl Jasmonate. Front. Plant Sci..

[26] Kendall S.L., Hellwege A., Marriot P., Whalley C., Graham I.A., Penfield S. (2011). Induction of Dormancy in *Arabidopsis* Summer Annuals Requires Parallel Regulation of *DOG1* and Hormone Metabolism by Low Temperature and CBF Transcription Factors. Plant Cell.

[27] Zeng W., Mostafa S., Lu Z., Jin B. (2022). Melatonin-Mediated Abiotic Stress Tolerance in Plants. Front. Plant Sci..

[28] Li H., Guo Y., Cui Q., Zhang Z., Yan X., Ahammed G.J., Yang X., Yang J., Wei C., Zhang X. (2020). Alkanes (C29 and C31)-Mediated Intracuticular Wax Accumulation Contributes to Melatonin- and ABA-Induced Drought Tolerance in Watermelon. J. Plant Growth Regul..

[29] Li H., Guo Y., Lan Z., Zhang Z., Ahammed G.J., Chang J., Zhang Y., Wei C., Zhang X. (2021). Melatonin Antagonizes ABA Action to Promote Seed Germination by Regulating Ca^2+^ Efflux and H_2_O_2_ Accumulation. Plant Sci..

[30] Li C., Tan D.-X., Liang D., Chang C., Jia D., Ma F. (2015). Melatonin Mediates the Regulation of ABA Metabolism, Free-Radical Scavenging, and Stomatal Behaviour in Two *Malus* Species under Drought Stress. J. Exp. Bot..

[31] Liu W., Zheng C., Chen J., Qiu J., Huang Z., Wang Q., Ye Y. (2019). Cold Acclimation Improves Photosynthesis by Regulating the Ascorbate–Glutathione Cycle in Chloroplasts of *Kandelia obovata*. J. For. Res..

[32] Ernst J., Nau G.J., Bar-Joseph Z. (2005). Clustering Short Time Series Gene Expression Data. Bioinformatics.

[33] Lefol Y., Korfage T., Mjelle R., Prebensen C., Lüders T., Müller B., Krokan H., Sarno A., Alsøe L., CONSORTIUM LEMONAID (2023). TiSA: TimeSeriesAnalysis—a Pipeline for the Analysis of Longitudinal Transcriptomics Data. NAR Genom. Bioinform..

[34] Haduch A., Bromek E., Kuban W., Daniel W.A. (2023). The Engagement of Cytochrome P450 Enzymes in Tryptophan Metabolism. Metabolites.

[35] Wu X., Ren J., Huang X., Zheng X., Tian Y., Shi L., Dong P., Li Z. (2021). Melatonin: Biosynthesis, Content, and Function in Horticultural Plants and Potential Application. Sci. Hortic..

[36] Jia K.-P. (2021). An Alternative, Zeaxanthin Epoxidase-Independent Abscisic Acid Biosynthetic Pathway in Plants. Mol. Plant.

[37] Yu Y. (2024). EjWRKY6 Is Involved in the ABA-Induced Carotenoid Biosynthesis in Loquat Fruit during Ripening. Foods.

[38] Dangol A., Shavit R., Yaakov B., Strickler S.R., Jander G., Tzin V. (2022). Characterizing Serotonin Biosynthesis in *Setaria viridis* Leaves and Its Effect on Aphids. Plant Mol. Biol..

[39] Gavyar P.H.H., Amiri H., Arnao M.B., Bahramikia S. (2024). Exogenous Application of Serotonin, with the Modulation of Redox Homeostasis and Photosynthetic Characteristics, Enhances the Drought Resistance of the Saffron Plant. Sci. Rep..

[40] Guo M. (2024). BAG8 Positively Regulates Cold Stress Tolerance by Modulating Photosystem, Antioxidant System and Protein Protection in *Solanum lycopersicum*. Plant Physiol. Biochem..

[41] Peng Y.-L., Wang Y.-S., Fei J., Sun C.-C., Cheng H. (2015). Ecophysiological Differences between Three Mangrove Seedlings (*Kandelia obovata*, *Aegiceras corniculatum*, and *Avicennia marina*) Exposed to Chilling Stress. Ecotoxicology.

[42] Wang Z., Yu D., Zheng C., Wang Y., Cai L., Guo J., Song W., Ji L. (2019). Ecophysiological Analysis of Mangrove Seedlings *Kandelia Obovata* Exposed to Natural Low Temperature at Near 30°N. J. Mar. Sci. Eng..

[43] Zhang H.-Y., Yue D.-F., Pan X.-J., Hao L.-L., Liu W.-C., Zheng C.-F. (2023). Effects of 5-HT on the Cold Resistance of Mangrove *Kandelia obovata* Seedlings. Ying Yong Sheng Tai Xue Bao J. Appl. Ecol..

[44] Li J., Chen S., Yin Y., Shan Q., Zheng C., Chen Y. (2023). Genome-Wide Analysis of bHLH Family Genes and Identification of Members Associated with Cold/Drought-Induced Photoinhibition in *Kandelia obovata*. Int. J. Mol. Sci..

[45] Fu J. (2022). Melatonin-Induced Cold and Drought Tolerance Is Regulated by Brassinosteroids and Hydrogen Peroxide Signaling in Perennial Ryegrass. Environ. Exp. Bot..

[46] Sanie Khatam A., Rastegar S., Hassanzadeh Khankahdani H. (2024). Modulating Cold Tolerance in Mexican Lime (*Citrus aurantifolia Swingle*): A Combined Approach Using Melatonin, Acetic Acid and Mannitol. Sci. Hortic..

[47] Liu L., Li D., Ma Y., Shen H., Zhao S., Wang Y. (2021). Combined Application of Arbuscular Mycorrhizal Fungi and Exogenous Melatonin Alleviates Drought Stress and Improves Plant Growth in Tobacco Seedlings. J. Plant Growth Regul..

[48] Imran M., Latif Khan A., Shahzad R., Aaqil Khan M., Bilal S., Khan A., Kang S.-M., Lee I.-J. (2021). Exogenous Melatonin Induces Drought Stress Tolerance by Promoting Plant Growth and Antioxidant Defence System of Soybean Plants. AoB Plants.

[49] Yu Y., Lv Y., Shi Y., Li T., Chen Y., Zhao D., Zhao Z. (2018). The Role of Phyto-Melatonin and Related Metabolites in Response to Stress. Molecules.

[50] Fujiwara T., Maisonneuve S., Isshiki M., Mizutani M., Chen L., Wong H.L., Kawasaki T., Shimamoto K. (2010). Sekiguchi Lesion Gene Encodes a Cytochrome P450 Monooxygenase That Catalyzes Conversion of Tryptamine to Serotonin in Rice. J. Biol. Chem..

[51] Lu H., Gao Q., Han J., Guo X., Wang Q., Altosaar I., Barberon M., Liu J., Gatehouse A.M.R., Shu Q. (2022). An ABA-serotonin Module Regulates Root Suberization and Salinity Tolerance. New Phytol..

[52] Greco M., Chiappetta A., Bruno L., Bitonti M.B. (2012). In Posidonia Oceanica Cadmium Induces Changes in DNA Methylation and Chromatin Patterning A GH3 Family Member, OsGH3-2, Modulates Auxin and Abscisic Acid Levels and Differentially Affects Drought and Cold Tolerance in Rice. J. Exp. Bot..

[53] Pan X. (2022). Transcriptional and Physiological Data Revealed Cold Tolerance in a Photo-Thermo Sensitive Genic Male Sterile Line Yu17S. BMC Plant Biol..

[54] Sulli M., Dall’Osto L., Ferrante P., Guardini Z., Gomez R.L., Mini P., Demurtas O.C., Aprea G., Nicolia A., Bassi R. (2023). Generation and Physiological Characterization of Genome-Edited *Nicotiana benthamiana* Plants Containing Zeaxanthin as the Only Leaf Xanthophyll. Planta.

[55] Yuan L., Shu S., Sun J., Guo S., Tezuka T. (2012). Effects of 24-Epibrassinolide on the Photosynthetic Characteristics, Antioxidant System, and Chloroplast Ultrastructure in *Cucumis sativus* L. under Ca(NO_3_)_2_ Stress. Photosynth. Res..

[56] Hu M.-J., Sun W.-H., Tsai W.-C., Xiang S., Lai X.-K., Chen D.-Q., Liu X.-D., Wang Y.-F., Le Y.-X., Chen S.-M. (2020). Chromosome-Scale Assembly of the *Kandelia obovata* Genome. Hortic. Res..

[57] Kim D., Paggi J.M., Park C., Bennett C., Salzberg S.L. (2019). Graph-Based Genome Alignment and Genotyping with HISAT2 and HISAT-Genotype. Nat. Biotechnol..

[58] Liao Y., Smyth G.K., Shi W. (2014). featureCounts: An Efficient General Purpose Program for Assigning Sequence Reads to Genomic Features. Bioinformatics.

[59] Love M.I., Huber W., Anders S. (2014). Moderated Estimation of Fold Change and Dispersion for RNA-Seq Data with DESeq2. Genome Biol..

[60] Tang D., Chen M., Huang X., Zhang G., Zeng L., Zhang G., Wu S., Wang Y. (2023). SRplot: A Free Online Platform for Data Visualization and Graphing. PLoS ONE.

[61] Du Z., You S., Zhao X., Xiong L., Li J. (2022). Genome-Wide Identification of WRKY Genes and Their Responses to Chilling Stress in *Kandelia obovata*. Front. Genet..

