# Supplementary material for: Serotonin Mitigates ColdStress-Induced Damage in Kandelia obovata Through Modulating the Endogenous Melatonin- and Abscisic Acid Biosynthesis
Source: Int J Mol Sci. 2025 Feb 14;26(4):1635. doi: 10.3390/ijms26041635 (PMC11855724; doi:10.3390/ijms26041635)
Supplement: Supplementary file 1 [file ijms-26-01635-s001.zip › ijms-3382902-supplementary.pdf]

Sample	Clean reads	Clean bases	Q20(%)	Q30(%)	GC content(%)	Both Surviving Read Percent(%)
CT-1	48281936	6.99E+09	98.51	95.46	45.3	98.12
CT-2	48627072	6.98E+09	98.38	95.1	45.3	98.18
CT-3	48309770	6.98E+09	98.31	94.92	45.33	98.09
LTS-1	48392956	7.01E+09	98.3	94.92	45.77	98.09
LTS-2	48016180	6.95E+09	98.35	95.03	45.74	98.12
LTS-3	47675960	6.92E+09	98.37	95.08	45.78	98.09
SER-CT-1	48013994	6.96E+09	98.53	95.48	45.71	98.16
SER-CT-2	47974112	6.96E+09	98.52	95.46	45.63	98.10
SERCT--3	48037106	6.93E+09	98.63	95.76	45.63	98.23
SER-LTS-1	48142160	6.95E+09	98.44	95.25	45.77	98.13
SER-LTS-2	47950994	6.9E+09	98.35	95.04	45.88	98.11
SER-LTS3	47492588	6.9E+09	98.59	95.65	45.67	98.20
Total	576914828					

Table S1. Statistical results of transcriptome

name	Primer pairs	
	Forward primer (5′-3′)	Reverse primer (5′-3′)
Raf	TGATGGTCGTAGTGGTAGAT	ACAGTTCCTACACTTTCCAC
CHLM	TGAGTTGGAATTATCCGGTG	CTTCCCCAATAGCAATCAGT
GGDR	TGGAGAAATGGGACAAGAAG	CACATCTCAACAAATGCCTC
PRK	GAAGTAACCCAACAAATGCT	TTCTGGCTTCTACTTCTACT
TRK	ACCTTGTCTTCTTCCTTACG	ATAGACTTCTCGACCAAGGA
MCF	TACAAGGGGATGGGAACTAA	GCCGAATAGTCTTGCTCTTA
PMP	AAGGAAGTGGAAGAAAGGAC	CAGTTTCTGTACCAACCAGA
LCACs	GATTTCTTCTGGAGGTGTGT	AGATAACAAGATGCGGACTC
18s RNA	GGGGCTCGAAGACGATCAGA	TTAAGCCGCAGGCTCCACTC

Table S2. Primer pairs used for RT-qPCR analysis

Specific name	Catalog number	Specifications
Plant ZEP ELISA KIT	YX-E28615	96T
Plant NCED ELISA KIT	YX-E28619	96T
Plant XD ELISA KIT	YX-E28636	96T
Plant AAO ELISA KIT	YX-E28630	96T
Plant TDC ELISA KIT	YX-200403P	96T
Plant T5H ELISA KIT	YX-200508P	96T
Plant SNAT ELISA KIT	YX-191421P	96T
Plant ASMT ELISA KIT	YX-011933P	96T
Plant TRP ELISA KIT	YX-201816P	96T
Plant TRY ELISA KIT	YX-201825P	96T
Plant SER ELISA KIT	YX-190518P	96T
Plant NAS ELISA KIT	YX-140119P	96T
Plant MT ELISA KIT	YX-132000	96T
Plant β-Carotene ELISA KIT	YX-E28616	96T
Plant zeaxanthin ELISA KIT	YX-E28618	96T
Plant V ELISA KIT	YX-E28617	96T
Plant Xanthixin ELISA KIT	YX-E28634	96T
Plant ABA ELISA KIT	YX-E21782	96T

Table S3. The catalog number, specific name and specification of the kits.

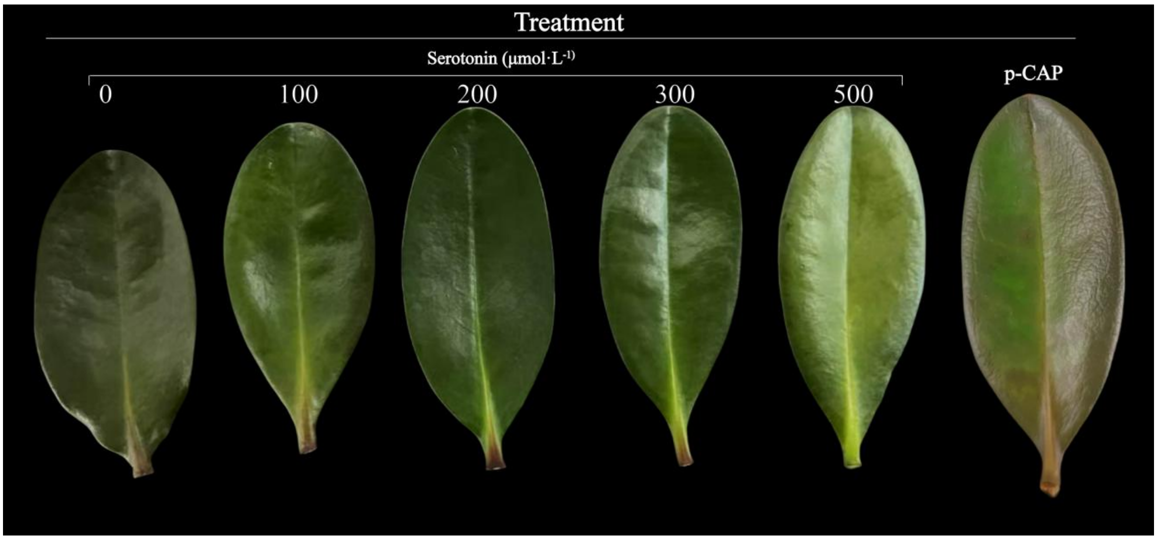

Figure S1. The leaves of *K. obovate* seedlings subjected to different concentrations of serotonin and p-CPA treatment under low temperature stress (6 °C/ -3 °C, day/night; 3 days).

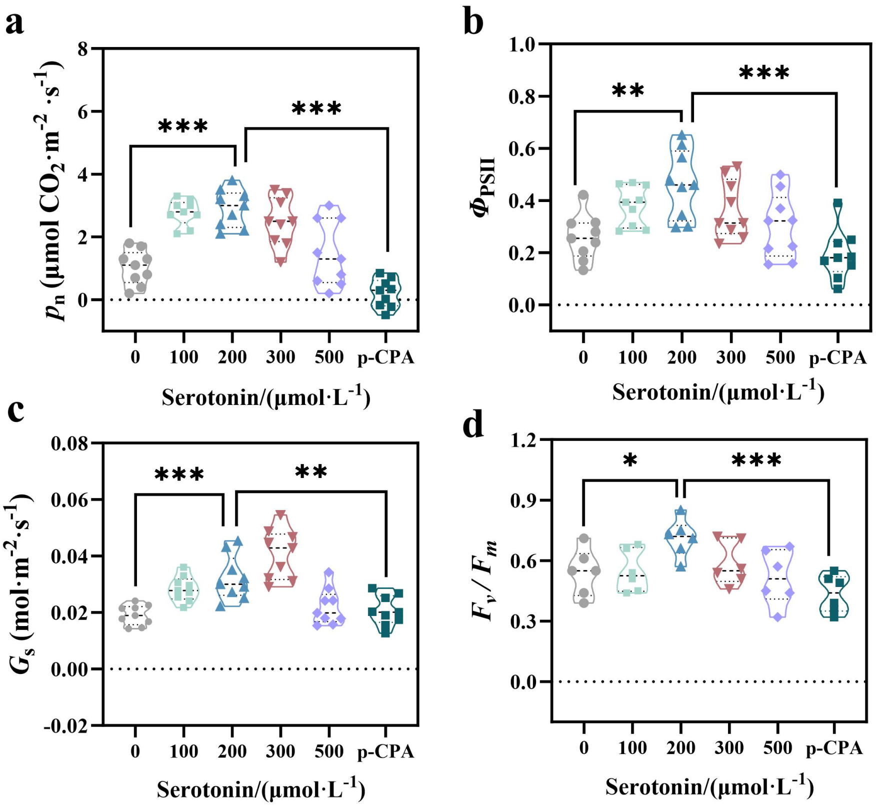

Figure S2. The alterations in photosynthetic parameters of *K. obovata* seedlings subjected to different concentrations of serotonin and p-CPA treatment under low temperature stress. (a) net photosynthetic rate (P_n), (b) the actual efficiency of PSII (Φ_{PSII}), (c) stomatal conductance (G_s) and (d) the maximum quantum efficiency of PSII (F_v/F_m). "**" and "***" indicate statistically significant at the P<0.01 and P<0.001 levels, respectively.

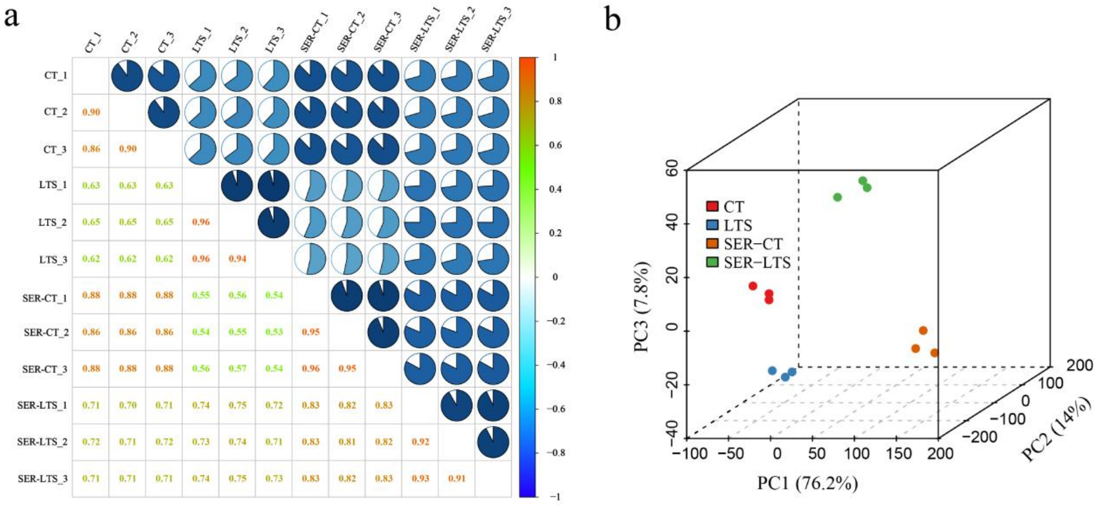

Figure S3. Transcriptome correlation and PCA analysis of different treatments of *K. obovata* seedling leaves. (a) Pearson's correlation coefficient analysis of gene expression values across all samples. (b) Principal component analysis (PCA) of gene expression values for all samples, with axes representing PC1, PC2 and PC3, and each color representing a different treatment. Three replicates were performed for each treatment.

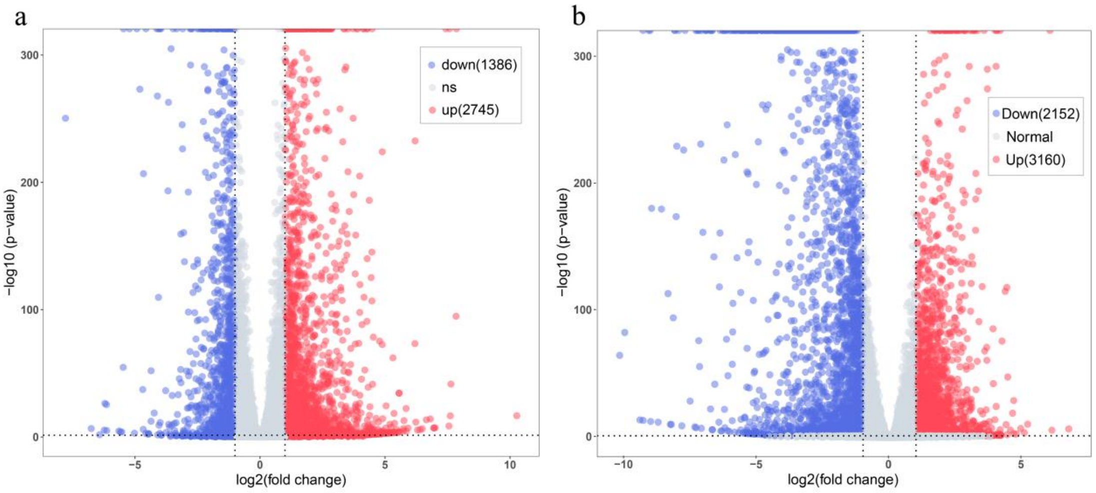

Figure S4. Differentially expressed genes (DEGs) of *K. obovata* seedlings with different combination of serotonin and low temperatures

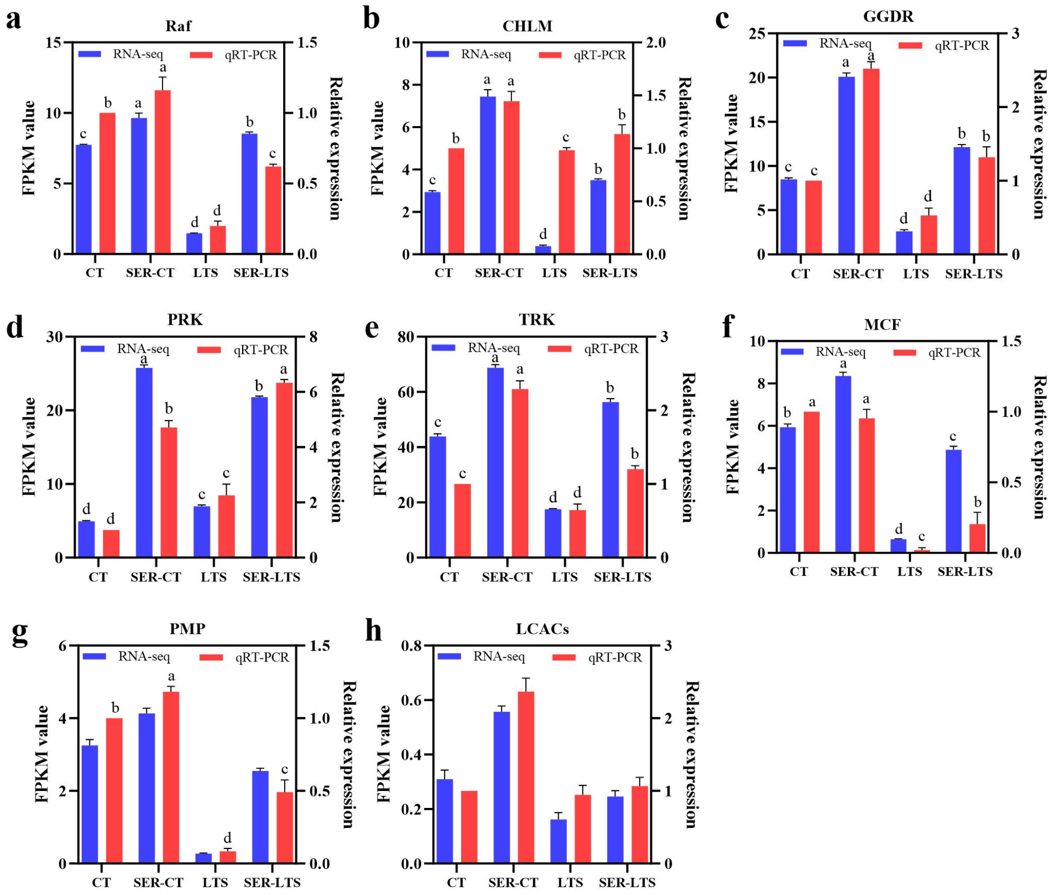

Figure S5. RT-qPCR analysis of genes related to photosynthesis, carbohydrate, amino acid, and nucleic acid metabolism in stressed *K. obovata* seedlings.

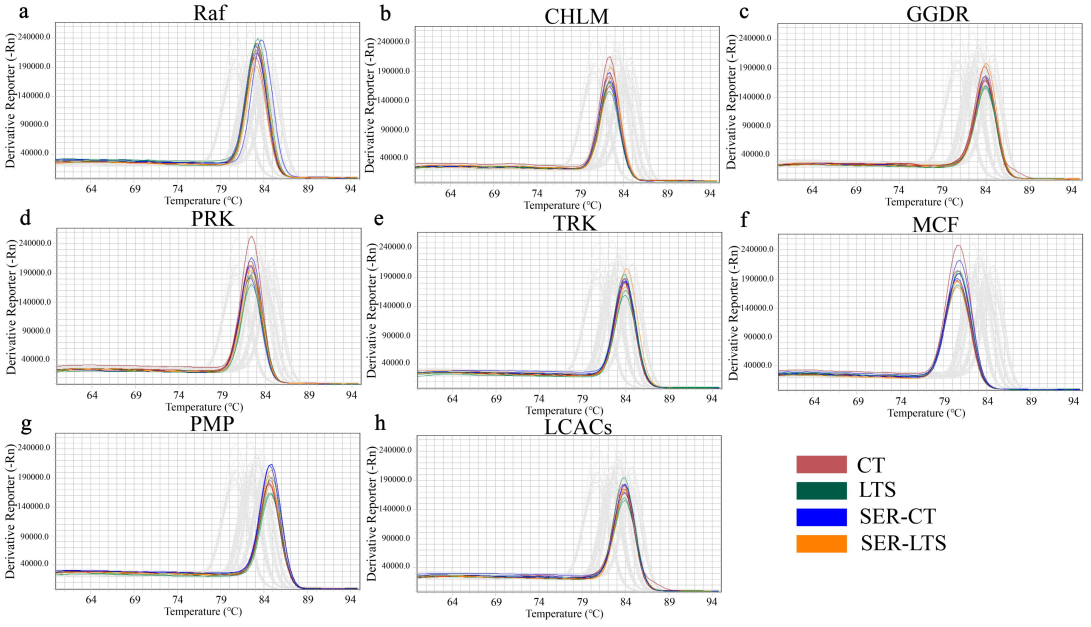

Figure S6. Melt curve plot of genes related to photosynthesis, carbohydrate, amino acid, and nucleic acid metabolism in *K. obovata* seedlings.
